# Implications of COVID-19 for future travel behaviour in the rural periphery

**DOI:** 10.1186/s12544-022-00547-0

**Published:** 2022-05-25

**Authors:** John D. Nelson, Brian Caulfield

**Affiliations:** 1 Institute of Transport and Logistics Studies, The University of Sydney Business School, The University of Sydney, Building H04, Sydney, NSW 2006 Australia; 2 Centre for Transport Research, Department of Civil, Structural and Environmental Engineering, Trinity College Dublin, The University of Dublin, Dublin 2, Ireland

**Keywords:** Rural, COVID-19, Public transport, Flexible transport, Innovative mobility

## Abstract

**Background:**

The design, management and operation of transport systems is a complex activity and this has only been exacerbated since the onset of the COVID-19 pandemic. Concern has been raised over the likelihood of the public transport sector surviving in some locations given the significant drops in patronage; this is especially so in rural environments where the existing provision was already limited. Furthermore, within the growing literature on the impact of COVID-19 on travel behaviour most of the focus is on urban areas with little documented experience of how rural travel behaviour has been impacted.

**Purpose:**

This paper investigates the impact of COVID-19 on the transport sector and travel behaviour in the rural periphery.

**Methods:**

Drawing on the work of the International Transport Forum (ITF) Working Group on Innovative Mobility for the Periphery, augmented by additional evidence and findings from the literature, this paper addresses three specific questions: Firstly, how COVID-19 has affected rural mobility. Secondly, how we can plan for sustainable rural transport solutions in the post-COVID world. Thirdly, the longer-term impacts of COVID-19 with implications for mobility.

**Results:**

There will be substantial impacts from COVID-19 on rural societies and while the short-term impacts have been negative, in the longer-term there may be opportunity for changed mobility behaviours (including in response to modified work and activity patterns). Evidence suggests that it would seem likely that there are opportunities to foster new rural mobility solutions to support sustainable mobility (including Mobility-as-a-Service) and counter the traditionally fragmented transport base; this will be important as we learn to live with COVID-19.

**Conclusions:**

While recognising the impact of changing funding priorities and the possible shift in economic activity as a result of the pandemic we conclude with suggestions for future rural transport policy.

## Introduction

The transport sector has been widely impacted by the COVID-19. In a public transport context, for example, [1] note the impacts arising from people being furloughed or working from home (WFH) and who thus have had no need to use public transport; furthermore, the need to self-distance and maintain good ventilation has posed particular challenges to the traditional operation and use of public transport (see for example [2]); and, in relation to aviation (which often provides “lifeline services” to remote locations), there has been a need to prevent travel so as to seek to halt the spread of COVID-19. Perhaps most dramatically, concern has been raised over the likelihood of the public transport sector surviving in some locations given the significant drops in patronage; this is especially so in rural environments where the existing provision was already limited. It is noteworthy that much of the reported impacts of COVID-19 on travel behaviour in the literature focuses on urban areas with relatively little documented experience of how rural travel behaviour has been impacted. This paper seeks to fill that gap.

This paper draws, in part, on the work of the International Transport Forum (ITF) Working Group on Innovative Mobility for the Periphery to address three specific questions: Firstly, the question of how rural mobility and travel behaviour have changed as a result of COVID-19, including some of the measures that public and shared transport operators have implemented in response. The influence of working from home (WFH) is also addressed. The second question focusses on planning for sustainable rural transport solutions in the “COVID-normal” world with a focus on the opportunities for more innovative solutions. The role of flexible and demand responsive transport services and the potential of Mobility as a Service (MaaS) for regional and rural areas is recognised. The final question addresses the longer-term impacts of COVID-19 and how transport planning can be made “fit for the periphery”. Implications of changing funding priorities are acknowledged.

The paper is organised as follows. The next section introduces the context of the study, recognising the varying definitions of “rural/periphery”, the factors influencing rural mobility and the importance of good stakeholder relationships in the rural transport system. The method adopted for this study is discussed in Sect. 3, followed by the findings (Sect. 4) which are structured according to the three specific questions identified above. Finally, conclusions are drawn.

## Context

In this paper, “periphery” is understood as “rural” and/or “remote” regions. However, definitions of “rural/remote” vary widely across the world. For example, [3], on behalf of the OECD, define “remote regions” as those regions where more than 50% of the population are located beyond a 60 min drive from urban areas of at least 50,000 people. They define “non-metropolitan regions” as regions where more than 50% of the population are located within a 60 min drive of urban areas of between 50,000 and 250,000 people. Both are considered to be within the scope of this paper.

A similar classification of the rural to urban continuum is offered by the Scottish Government [4] which identifies: Accessible Small Towns (settlements of 3000–9999 within a 30-min drive time of a settlement of 10,000 or more people); Remote Small Towns (3000–9999 within a drive time of over 30 min to any settlement of 10,000 or more); Accessible Rural Areas (areas with a population of < 3000 within a 30-min drive time of a settlement of 10,000 or more); and Remote Rural Areas (areas with a population of < 3000 and with a drive time of over 30 min to a settlement of 10,000 or more.

In Canada, a rural area is defined broadly as any area that is not a population centre as per the census [5]. Examples include areas with a population less than 1000, agricultural lands, and remote/wilderness areas. Small (1000–29,999), medium (30,000–99,999) and large urban (> 100,000) population centres are also defined.

The Australian Rural, Remote and Metropolitan Area’s ‘Index of remoteness’ [6] is considered more comprehensive since it is based on distance to service centres as well as a measure of ‘distance from other people’. However, it is acknowledged that these distance-based measures do not explicitly take account of other aspects such as the cost of travel options, access to publicly available modes of travel, rates of car ownership, or the safety of travel options.

A lack of opportunities to fulfil requirements of mobility in the periphery impacts on people’s ability to access services, and hence on people’s quality of life [7]. Ideally, rural areas would be served by a co-ordinated transport system capable of making best use of the available transport resource, which may include bus services, taxis, Community Transport, education, health and social service transport as well as various car-based options such as car clubs (also widely known as car sharing) and voluntary car services. It has been demonstrated that scope exists for releasing otherwise unused capacity where it may be possible to relax eligibility criteria for certain transport services [8].

One way to advance the mobility agenda in remote and rural areas is to maintain a good understanding of the stakeholders involved and their roles. This may include representatives of policy makers, transport operators and co-ordinators, providers of finance, trade associations and (importantly) passenger groups. The importance of good stakeholder relationships will be demonstrated in the findings section of this paper.

Finally, in terms of context, it is remarkable to note the paucity of specific policy for mobility in rural areas. Mounce et al. [7] note that across the European Union, for example, most of the 27 countries do not have any policy at all and that no country has specified levels or obligations in terms of rural mobility. Further information is available in [9]. This is particularly concerning when faced with a significant external shock such as COVID-19.

## Method

The material presented in this paper draws in part from the outcome of the ITF Working Group on Innovative Mobility for the Periphery which was active between October 2020 and January 2022.^1^ The objective of the Working Group was to investigate rural mobility innovation across the globe covering the whole spectrum of mobility systems implemented or in pilot phases, from national or regional authority initiatives to projects by individual transport operators and local community groups. As part of its programme of work the Working Group conducted a survey amongst its member countries. The survey was administered by the ITF secretariat and recipients included representatives of transport ministries, agencies and publicly funded research centres.

The objective of the survey was to learn more about current and recent mobility innovations for rural, peripheral and remote areas. After seeking definition of peripheral, remote and rural in a specific country context information was sought about central or regional government objectives and targets regarding mobility in peripheral, rural and remote areas; whether accessibility is measured; the organization, financing and procurement of transport services; regulations or rules affecting innovative mobility services; laws, or guidelines addressing data governance related to new mobility services; and the levels of government or authorities responsible for the planning and implementation of traditional and new mobility services in rural areas. Information was also sought on any barriers to innovation and measures being implemented to address these. Respondents were encouraged to include details (including supporting documentation) of all relevant initiatives and projects relevant to their country that they were aware of and to detail any pilot projects.

The survey included two questions specifically relating to COVID-19 and answers to these are included in the findings (Sect. 4).

- How has the COVID-19 pandemic affected public transport specifically in peripheral, rural and remote areas?
- With respect to any pilot projects in rural areas—How has COVID-19 affected the service?

The survey ran from January to April 2021 and responses from 13 different countries^2^ were received. Findings from the survey relevant to the impact and implications of COVID-19 are discussed in the next section (this is particularly relevant to the first of our specific questions for discussion) supplemented by discussions that took place within the Working Group and literature analysis. The outcome of the Working Group is reported in [10].

## Findings

This section addresses three specific questions relevant to the implications of COVID-19 for rural mobility:

- How COVID-19 has affected rural mobility
- How we can plan for sustainable rural transport in the post-COVID world
- The longer-term impacts of COVID-19 with implications for mobility

### How COVID-19 has affected rural mobility

It is relevant to note that much of the reported impact of COVID-19 on travel behaviour focuses on urban areas (see for example [11–13] and there is relatively little documented experience of how rural travel behaviour has been impacted. Several country-level studies have been conducted, including for Germany [14], Poland [15], Sweden [16] and Australia [17, 18], although the focus is predominantly urban/metropolitan. This pattern was confirmed by the ITF Working Group survey on rural mobility (referred to in Sect. 3) which revealed a lack of rural specific data for several countries including Germany, Denmark, Mexico, Norway and Poland.

**Fig. 1:**
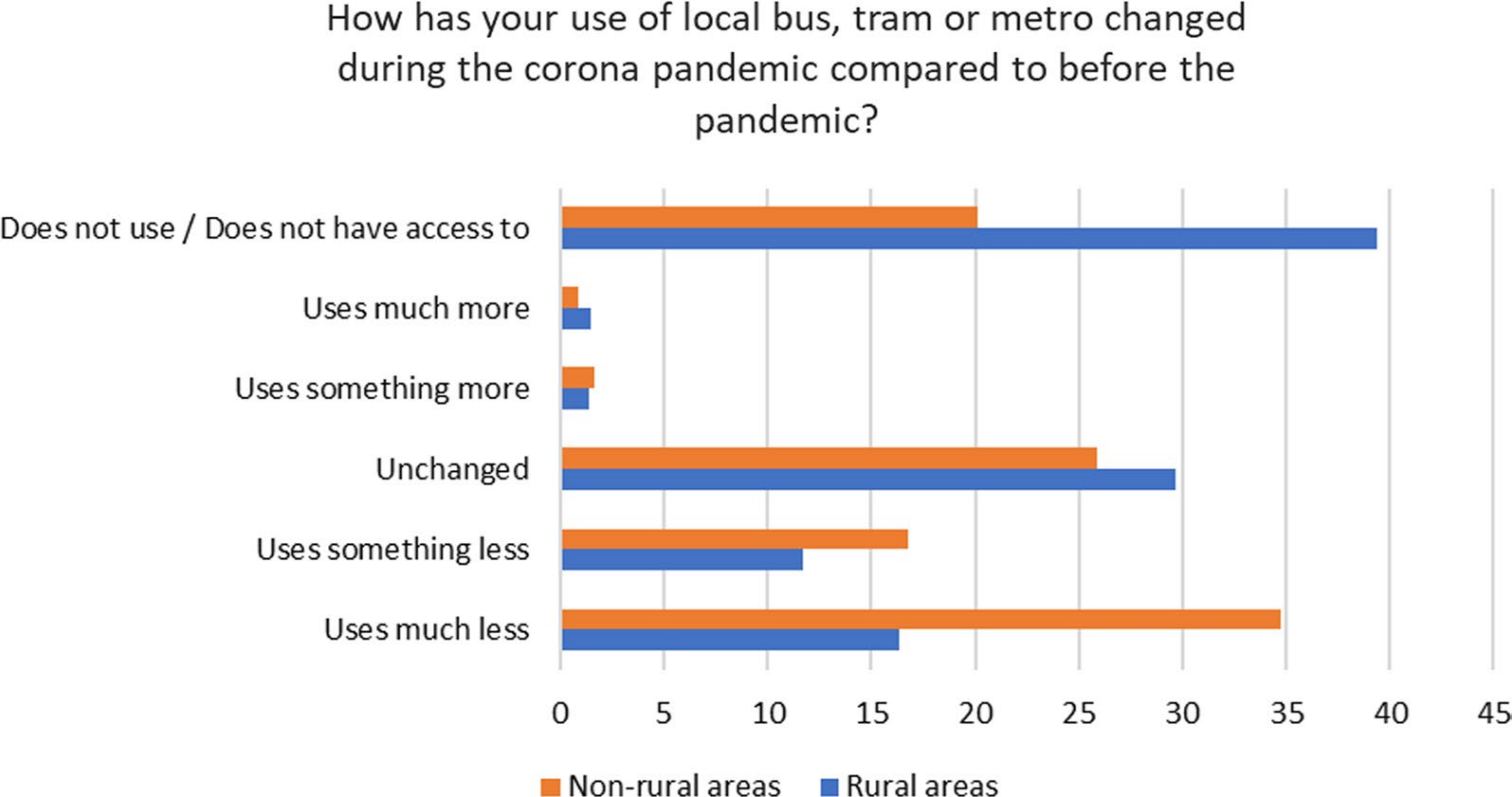
Use of local public transport in Sweden before and after the pandemic (% of responses) ( Source: ITF survey, 2021)

Findings from the ITF Working Group survey indicated that in Sweden (as in many countries) there are few statistics available on travel activities in the light of COVID-19 based on geographical location. However, an on-going study on people´s use of public transport during the pandemic [19] shows that people in rural areas report less changes in travel with local public transport compared to people in small, medium and large urban areas (see Fig. 1). Data were collected in June 2020 from a questionnaire; there were 152 responses from rural areas and 852 from non-rural areas. This result can perhaps be explained by the fact that there are fewer transport alternatives and fewer people in rural areas.

The New Zealand survey response reported that Waka Kotahi (the NZ Transport Agency) has undertaken a COVID-19 tracking project that sought to understand: (1) how travel is changing and evolving in response to the virus on a weekly basis (in aspects such as trip frequency and journey type changes), and (2) why travel is changing and evolving in response to COVID-19, by capturing perceptions/attitudes towards COVID-19 and travel options. From this research (May 2020) there is a limited amount of analysis that has been undertaken for rural areas [20]. A relevant finding is that those living in rural areas are much less likely to say that they can easily get to where they need to during lockdown, with suburbanites most likely to agree that they can meet their mobility requirements. Interestingly, rural residents were more likely to disagree that citizens were looking out for each other than their urban counterparts. At a more general regional level public transport usage declined in periods of lockdown with people stating that they were travelling less in general [21].

In Japan in rural areas pre-COVID-19, people commonly reported feeling uneasy about the decline of public transport and wanted good public transport to maintain their quality of life. Elderly people living in rural areas in particular were very worried that it would be difficult to get around if they cannot drive [22]. The survey response confirmed that these issues have been exacerbated by the pandemic.

More anecdotally one can identify cases of how rural mobility has been impacted by COVID-19. Public and community transport (CT) plays a vital role in rural areas. Older people, and those that don’t have access to a car, rely on these services for health, wellbeing and social connection and as a social service supporting independent and healthier lifestyles [23].

**Fig. 2:**
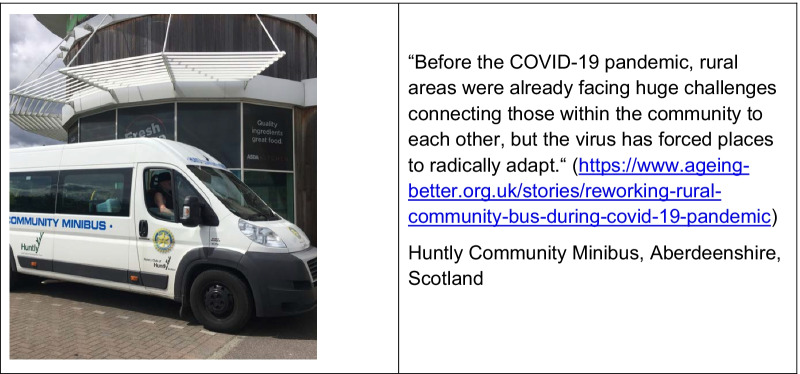
Huntly community minibus

Before the outbreak of the COVID-19 pandemic, the Huntly Community Minibus in Scotland (Fig. 2) was a very busy service, running regular outings for care home residents and many community groups. During the pandemic the bus has been a lifeline in assisting people with their shopping, hospital appointments and providing essential human interaction. The Community Bus is one of the few remaining transport options for rural residents without their own vehicle and is the only CT locally with wheelchair access. With the onset of the pandemic the regular social activities the bus was used for had to cease and it was decided that the bus would be available on a taxi-like basis—travellers call to request a pickup and arrange a suitable time when the bus is available. Donations are welcome but the service is free to use. Adaptations were made to ensure the bus was complying with official guidance on physical distancing and cleaning. The bus is available for anyone to use but the main customers are older people who don’t have access to a car. It has become a vital service for those who are otherwise cut off in their rural communities.

In Mexico, according to the survey, information retrieved from news reports and other informal sources suggests that in the municipality of Los Choapas, Veracruz, rural transport runs have decreased by up to 60% as a result of the pandemic, due to the strict control of the communities over who enters and leaves them. In the municipality of Mocorito, Sinaloa, several rural transport routes could disappear altogether due to the zero daily passengers. The cancellation of face-to-face classes in schools caused the flow of passengers between the city of Mazatlán, Sinaloa and the rural area to the south of this state to fall more than 50%.

In the Canadian context, as reported in the survey, publicly available data from Statistics Canada and other surveys mostly relate to the COVID-19 impact on urban public transport. However, there is evidence of service closures that primarily impact rural communities. For example, Greyhound Canada, an intercity bus service that served rural, remote, and Indigenous communities, terminated its services on May 13, 2021, having suspended their services in May 2020 with the onset of the pandemic. The bus company has cited a drop in ridership of 95% due to the pandemic as one of the rationalizing factors for their end of service. News reports have mentioned the isolating effect this loss of service will have on rural communities [24]. Greyhound used to be Canada’s largest interprovincial bus operator but they had been reducing their services slowly over the last few decades. However, the US survey response indicated that there is evidence that rural and suburban bus systems experienced much higher ridership levels than their urban peers.

Despite the numerous instances of service cessation amongst shared transport services around the world, stories of adaptation and resilience abound. For example, in order to assist with COVID-19 and reduced vehicle capacity, one demand responsive transport (DRT) operator in New South Wales started to pick up parcels for the price of a fare. This has been popular with the elderly and vulnerable customers. Similarly, in the UK the CT sector has been given permission to distribute goods and packages. In Canada, rural transportation and mobility services have also adapted in the context of the COVID-19 pandemic. The Rural Transportation Association (RTA) in Nova Scotia, Canada supports community-based transportation in the region. In partnership with the Government of Nova Scotia, they have been offering round trips for a CAD 5 flat rate to vaccination clinics. Innisfil, a rural town in Ontario, Canada, currently operates on-demand, door-to-door transportation services in partnership with Uber. During the pandemic, the Town of Innisfil created the Essential Trips Assistance Program to offer limited free trips to areas including grocery stores, pharmacies, and health centres [25]. In Timaru, New Zealand, MyWay by Metro [26], a trial on-demand shuttle bus service has operated as usual during lockdown. During the COVID-19 period, the service found there was a higher demand for the service.

Another significant consequence of COVID-19 which cannot be ignored is the influence of working from home (WFH) on transportation demand. The dramatic increase in people WFH has been hugely successful in showing that it is not necessary for many to travel each day, although WFH under lockdown is not the same as WFH by choice. It is an example of a non-transport policy to help solve a transport problem. But some aspects still need to be validated was productivity higher, is worker stress increased? Nevertheless, it is clear that some degree of working from home is likely to be here to stay and to be encouraged. Studies (e.g. [17]) suggest that 30–40% of those who can, may work from home in the future for between 1 and 2 days per week. Is this an opportunity or a threat for rural areas? If home working becomes a long-term pattern, people may be encouraged to move from cities to the countryside since these new residents can provide a much-needed injection of cash into rural areas, but they will relocate only if they can find adequate infrastructure and community services in those areas [27]. Evidence from Statistics Canada [5] suggests that COVID-19 may have accelerated the movement of individuals to suburban and rural areas. Additionally, news reports have cited a real estate boom throughout Canada during the pandemic, including in cottage country and smaller towns. This may be evidence of a trending urban exodus due to an increase in teleworking, amongst other trends.

### How we can plan for sustainable rural transport in the post-COVID world

Some of the ways in which COVID-19 has highlighted the need for better rural transport are discussed by [28], who comments that travel needs have evolved in response to COVID-19, with the rise in home working, concerns around conventional public transport and interest in relocating from cities to rural and island areas. Thus, despite the challenges, there is not only an opportunity but a demand for a new way to travel in rural areas which better considers the needs of local communities. This should entail taking a closer look at the requirements of travellers (resident, seasonal, business recreational, etc.) and providing them with mobility solutions appropriate to their needs.

In planning for public transport in the post-COVID world a number of observations (based on [29]) can be made:

1. Given the important role of public transport as an essential service this is a good reason for using public money to finance the system.
2. The need/desire to keep physical distancing can perhaps induce a revision of what is considered an acceptable capacity in public transport vehicles.
3. The pandemic has also induced more active travel as a replacement for short-distance travel by public transport. Improved conditions for the active modes, including in rural areas where e-bikes could play a role, will support network design recommendations concerning greater distances between stops, faster and more direct and frequent services along key routes.
4. The travel patterns being exhibited in the “current normal” imply that public transport customers have become more adaptable and less predictable and with greater flexibility as to where and when they work. Might a more personalised transport offer with elements of flexibility be seen as more attractive?
5. Building on point 4, in some locations it may be more efficient to run On Demand instead of fixed route services on a larger scale to reflect this “new normal”.

Nelson and Wright [30] document experience with flexible transport services (FTS) in rural areas where evidence shows that FTS can be deployed as an effective component of an integrated transport offering. It is relevant to note that there are some positive findings about the experience with FTS during the pandemic. In a case-study of On Demand Transport (ODT) in regional New South Wales (NSW), [31] notes that on-demand patronage in NSW at September 2020 (6 months into the pandemic) was at 74% of passenger numbers in February, while fixed route bus was at 54%.

**Fig. 3:**
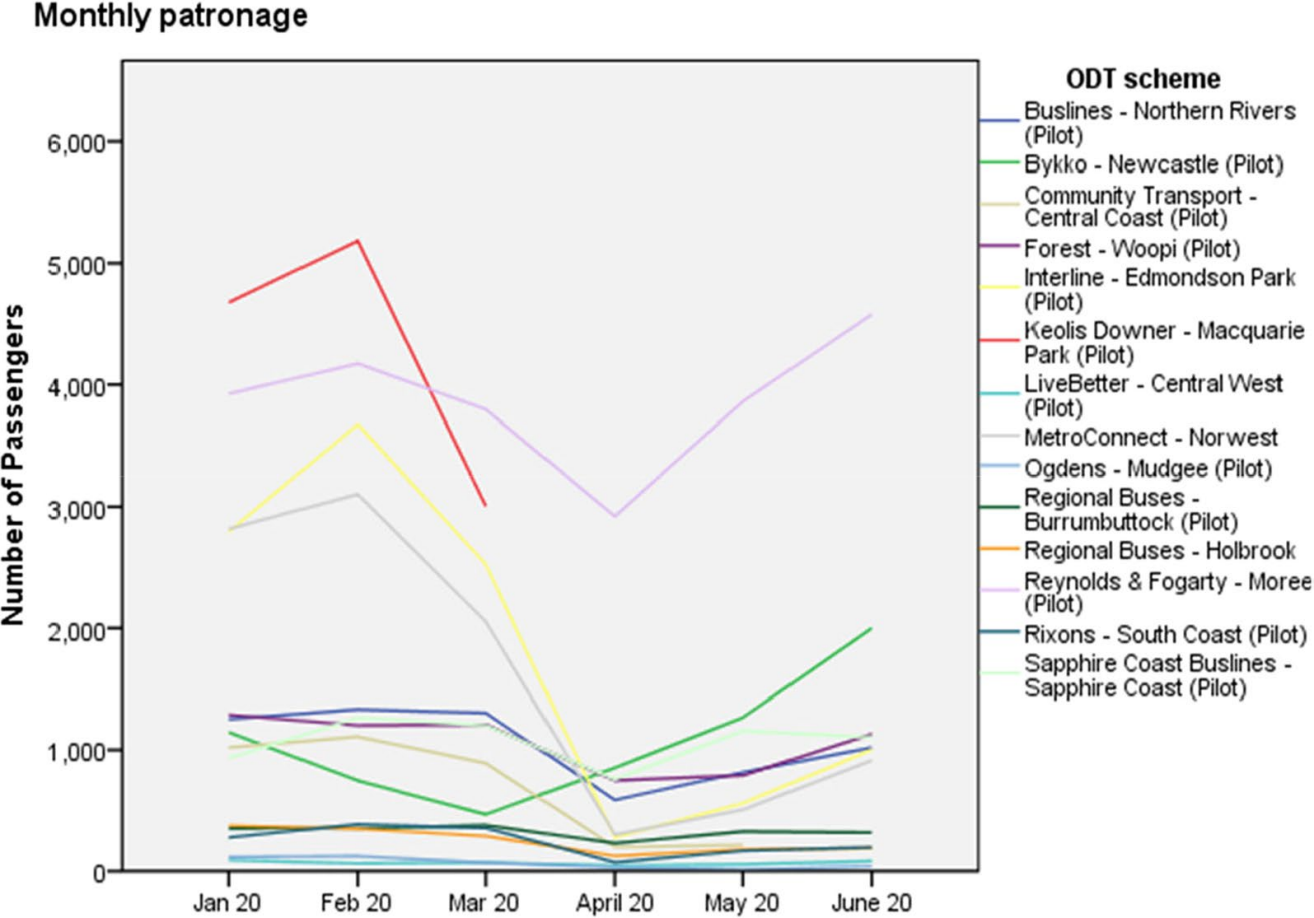
ODT patronage trends, Jan–June 2020 ( *Source* TfNSW Open Data Hub). *Note* The two largest schemes by patronage are excluded: Keolis Northern Beaches with monthly patronage in June around 10,000; pre-COVID-19 levels were around 18,000 passengers / month; and Cooee—The Ponds with monthly patronage in June around 5000 and pre-COVID-19 levels of around 10,000. Macquarie Park ceased operation in March 2020 as planned

Figure 3 shows patronage experience for the NSW schemes for the first 6 months of 2020, which includes the 6-weeks lockdown which began in late March and shows the extent to which ODT has “bounced back”. During lockdown, ODT (like other forms of public transport), was characterised as an essential service. With ODT the ability to book ahead ensures that physical distancing is maintained (and vehicles are larger than those of taxis and TNCs) and ODT through its booking and confirmation of pick-up and set down ensures contact tracing where required [31]. Cashless payment (where available) adds confidence.

Perhaps these trends are not so surprising given that the ODT offer is traditionally predicated as a personalised transport service. Another important trend shown in Fig. 3 is that the rate of recovery has been greater for some regional and rural schemes (compared to metropolitan areas) as witnessed by the Moree pilot (now permanent) and the Woopi Connect service at Coffs Harbour. There may also be a service design issue here since the best performing service in terms of recovery (Moree) is an area-based rather than a feeder to fixed route service which typically is designed for commuters, who have a reduced need to go into the office (the latter point is demonstrated by the minimal recovery of patronage figures on the Edmondson Park and Norwest services which are peak hour only first/last mile services). Operators in other jurisdictions (for example, South Australia) reported similar experiences and findings confirm the importance of locational context on ODT patronage [32].

An active Rural MaaS agenda continues to develop, although the debate around MaaS has been hampered by the lack of a suitable benchmark definition [33].^3^ Rural MaaS (like urban MaaS) will need to overcome the effects of COVID-19 and the growing concern that the considerable subsidy required for Public MaaS (the most likely option for rural areas where the integrator role is either the public authority or the public transport operator) is only likely if the take-up is such that sustainability goals and objectives will be met [33].

Eckhardt et al. [34] have advocated for rural MaaS pilots based on public private partnerships between local and regional stakeholders such as end users, transport service providers, digital service providers and relevant tiers of government. Recent experience from Finland, reported in the ITF Working Group survey, shows that the global pandemic has made the implementation of the MaaS experiments significantly more difficult by bringing uncertainty to the continuation of experiments and delays to the development of MaaS solutions. In particular, the pandemic has interrupted the combination of transport services which is so essential to MaaS solutions. Furthermore, the pandemic may result in changes both in citizen’s attitudes towards passenger transport and in the willingness of transport operators to invest in new solutions. Experiences are similar in Sweden [35].

Critically, rural and regional areas are quite distinctive from urban areas. As a result, any MaaS provision is unlikely to be built on a strong regular route-based public transport offer, and therefore car-based solutions are important to include in the mix with potentially more flexible forms of public transport services. Hensher et al. [36] argue that it is time to rethink the role of the car in a MaaS offering and suggest that we should consider the appeal of including electric cars as part of the sharing service, which they call electric car sharing as a service (ECSaaS). Such a scheme could work well in a Rural/Regional setting where having public transport as the centre of an eMaaS offering is less likely. In a similar vein, [35] describe recent experience from 5 rural MaaS pilots in Sweden noting the prevalence of car-based modes in the offer (three of the pilots were disrupted or paused because of COVID-19).

Some of the challenges are well summarised by the following quote: “Rural MaaS doesn't look exactly the same as MaaS implemented in or planned for urban areas around the world. The objective of MaaS in rural areas is to increase efficiency and utilization rates of shared transportation options, as well as maintaining sufficient service levels and improving accessibility. Using Mobility Equity Indicators, it is clear that certain services should be given higher priority in a rural setting than others. For example, on-demand rideshare programs are much more effective in rural communities than bikesharing programs, and thus should be given higher priority” [37].

Nelson and Wright [30] note that more research is needed on how to best include non-timetabled (flexible) public transport services within MaaS. However, this is more likely for Microtransit solutions in urban areas where an ‘always available’ service exists. It will be necessary to consider the effect that COVID-19 will have on market demands and operator supply and whether this will lead to more or less interest/need for FTS solutions. For example, it may be that passengers spread in small groups across smaller vehicles (FTS) are better than larger numbers of passengers physically distanced on fewer large vehicles (conventional buses) at a greater cost of provision but with greater flexibility of operation. Bruzzone et al. [38] in a study from the town of Velenje in rural Slovenia describe a study which explores the potential integration of an electric bike-sharing system and a semi-flexible demand-responsive transport system. They suggest that with comparable level of funding to that of conventional bus services the service level could be surpassed.

### The longer-term impacts of COVID-19 with implications for mobility

The longer-term impacts of COVID-19 on mobility in rural areas is unknown. Cabras [27] notes that rural communities, in particular, will be hit hard. The closure of local hubs such as pubs and community centres in rural and remote areas has been a trend in recent years and this has been exacerbated by the pandemic. Furthermore, the impact of measures imposed by governments to contain contagion—such as the closure of non-essential businesses and the instruction to work from home where possible have been felt particularly strongly in rural communities.

It will be interesting to see the long-term impacts of COVID-19 on the development of business models and tech with respect to ride-share and on-demand services, and especially whether changes in demand/social licence impacts availability and or innovation in this area.

It should also be recognised that it is harder to work from home in many rural areas on account of internet bandwidth, an issue that has been recognised for many years. Farrington et al. [39] identified a two-speed Britain in which over 1 million people were potentially excluded from, or at best found it challenging to participate in, what is generally regarded as ‘normal’ online social, commercial, creative and civic life, because they live in deep rural areas. In the US nearly one-third of rural residents do not have access to high-speed internet, and of the 24 million people who lack even internet access, 80% of them are in rural areas [40]. In the Canadian context, data from 2017 [41] shows that 63% of rural households and 76% of Indigenous communities did not have access to 50/10 Mbps Internet speeds, compared to only 3% of urban households.

**Fig. 4:**
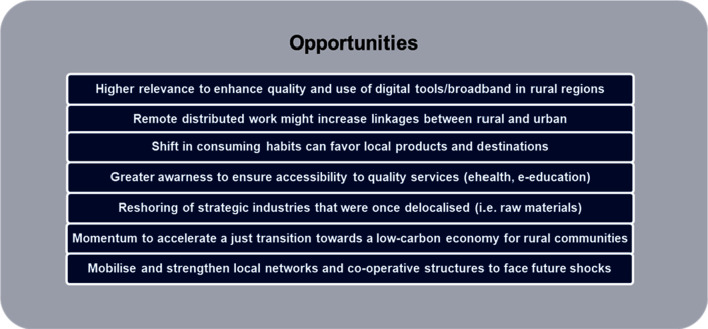
Opportunities emerging with the COVID-19 crisis [42]

More positively, there is evidence that the move to an online world has resulted in new business opportunities with a greater emphasis on bringing services to people (home delivery, telehealth, etc.), rather than people needing to travel to services, and a more effective provision of some community services. Some community groups (e.g., churches and clubs) have found that their activities have been invigorated by a strengthened online presence, although one must be mindful of demographic factors (e.g., rural populations are often older) that can contribute to the (so-called) digital divide as well as digital literacy. Certainly, COVID-19 has shown that individual behaviour can be substantially modified and travel substituted through technological innovations [38] and this provides an important foundation for the future of rural communities. The digital opportunities for rural areas have been identified by [42] and are summarised in Fig. 4.

Many rural areas were already vulnerable with respect to Government priorities and there is a danger that this remains the case. In the pre-pandemic era, the UK Government had stated that given the low population density (and hence low profitability) of rural areas, it is a challenge for the market to provide efficient, sustainable transport solutions [43]). But is this the correct objective when many basic mobility needs continue to be unmet in many rural environments? Related concerns have been raised in the US by TRIP (The Road Information Program) who estimated (in June 2020) that State transportation revenues could decrease by 30% (approx. USD 50 billion) over 18 months due to reduced vehicle travel as a result of COVID-19, leading to delays to repairs and improvements to the rural transportation system (specifically roads and bridges) [44].

The effect of loss of revenue due to cessation of international travel is another pressing concern. For example, international visitor arrivals to Australia in January 2022 were down 93% relative to 2 years earlier with knock-on effects for tourism revenue which has direct implications for regional and rural locations which very often depend on the revenue generated by tourism. This is not a shortfall that can be made up by increased domestic spend [45].

## Conclusions

This paper has sought to investigate the impact of COVID-19 on the transport sector and travel behaviour in the rural periphery. It is clear that there will be substantial impacts from COVID-19 on rural societies and while the short-term impacts have been negative, in the longer-term there may be opportunity for changed mobility behaviours. Evidence suggests that it would seem likely that there are opportunities to foster new rural mobility solutions to support sustainable mobility and counter the traditionally fragmented transport base; this will be important as we learn to live with COVID-19. Also, it is possible that WFH may stimulate some urban–rural migration. The COVID-19 era has led to calls for a more “responsible” transport agenda characterised by a greater role for individual choice and actions in collectively delivering socially desired outcomes [46]. Budd and Ison [46] go on to note that the spread of Uber, EVs, electric bikes and e-scooters will increasingly provide the context in which Responsible Transport will develop, and while they are writing in an urban context these ideas should be seen as also relevant to the rural environment.

A number of suggestions for future rural transport policy arise from this study and are summarised below:

- National/regional level transport data collection should give greater attention to the rural context.
- Given that the underlying fragility of the rural public transport network has been exacerbated by the pandemic greater resources should be directed towards strengthening essential transport services.
- The Community Transport sector should be recognised for the “lifeline” services they provide to rural communities.
- Given that the pandemic may result in changes in attitudes towards traditional passenger transport more attention should be given to flexible and responsive forms of transport (including taxi services) and to MaaS-type solutions in rural environments which also recognise the role of the private car.
- Investment in digital infrastructure in rural areas should go hand in hand with investment in physical transport infrastructure.
- WFH should be recognised as a demand management tool, although further work is needed to identify the kinds of benefits that may accrue to rural societies.
- Remote working hubs might be one tool to revitalise rural areas, giving workers the ability to move out of urban cores to work on a full-time or part-time basis from a remote working hub.

## Data Availability

Data were obtained from a survey organised by the ITF Working Group on Innovative Mobility for the Periphery which ran from January to April 2021. Relevant findings from the survey were supplemented by literature review and discussions within the Working Group.

## Footnotes

1 The following countries participated in Working Group activities: Australia, Austria, Argentina, Canada, Chile, Denmark, Finland, France, Germany, Ireland, Japan, Latvia, Mexico, New Zealand, Norway, Poland, Sweden, the United Kingdom and the United States.

2 Responses were received from the following countries: Argentina, Canada, Denmark, Finland, France, Germany, Japan, Mexico, New Zealand, Norway, Poland, Sweden and the USA.

3 Recognising the need for a clarifying definition of what constitutes MaaS (and what does not) and to develop a reference benchmark [33] offer the following definition: “MaaS is a framework for delivering a portfolio of multi-modal mobility services that places the user at the centre of the offer. MaaS frameworks are ideally designed to achieve sustainable policy goals and objectives. MaaS is an integrated transport service brokered by an integrator through a digital platform. A digital platform provides information, booking, ticketing, payment (as PAYG and/or subscription plans), and feedback that improves the travel experience. The MaaS framework can operate at any spatial scale (i.e., urban or regional or global) and cover any combination of multimodal and non-transport-related multi-service offerings, including the private car and parking, whether subsidised or not by the public sector. MaaS is not simply a digital version of a travel planner, nor a flexible transport service (such as Mobility on Demand), nor a single shared transport offering (such as car sharing). ‘Emerging MaaS’ best describes MaaS offered on a niche foundation. This relates to situations where MaaS is offered on a limited spatial scale, to a limited segment of society or focused on limited modes of transport. The MaaS framework becomes mainstream when the usage by travellers dominates a spatial scale and the framework encompasses a majority of the modes of transport.”
